# Ewing Sarcoma of the Maxilla: An Uncommon Anatomical Presentation in a Young Patient

**DOI:** 10.7759/cureus.100129

**Published:** 2025-12-26

**Authors:** José R Velázquez-Soto, Daniel Rosales-Rosales, Carlos A Jaimes-Loreto, Lizeth R Ríos-Bañuelos, Rachel V Cárdenas-Cabral, Quitzia L Torres-Salazar

**Affiliations:** 1 Oncology, Centro Estatal de Cancerología del Estado de Durango, Durango, MEX; 2 Internal Medicine, Instituto Mexicano del Seguro Social, Durango, MEX; 3 Internal Medicine, Universidad Autónoma de Durango, Durango, MEX; 4 Pathology, Centro Estatal de Cancerología del Estado de Durango, Durango, MEX; 5 Oncology, Universidad Juárez del Estado de Durango, Durango, MEX; 6 Biomedical Sciences, Universidad Juárez del Estado de Durango, Durango, MEX

**Keywords:** ewing sarcoma, head and neck neoplasms, immunohistochemistry, maxilla, multimodal therapy

## Abstract

Ewing sarcoma (ES) of the maxilla is an exceptionally rare entity that can mimic odontogenic or inflammatory conditions, often resulting in diagnostic delay. We report the case of a 23-year-old female who presented with a painless, progressively enlarging swelling of the anterior maxilla. Imaging demonstrated an expansile osteolytic lesion with cortical disruption. Histopathologic evaluation revealed a small round cell neoplasm, and immunohistochemical analysis showed diffuse membranous expression of cluster of differentiation 99 (CD99) and focal nuclear positivity for Friend leukemia integration 1 (FLI-1), supporting the diagnosis within the ES family of tumors in the appropriate clinicoradiologic context. The patient underwent subtotal maxillectomy with intraoperative margin assessment, achieving complete surgical excision with histologically negative margins, followed by adjuvant chemotherapy with epirubicin, cisplatin, and ifosfamide, as well as localized radiotherapy. At 18 months of clinical and radiologic follow-up, there was no evidence of local recurrence, with preserved oral function and satisfactory aesthetic outcomes on clinical evaluation. This report highlights the importance of considering malignant small round cell tumors in rapidly enlarging maxillofacial lesions and emphasizes the role of multidisciplinary management in achieving sustained local disease control while preserving postoperative function.

## Introduction

Primary bone tumors represent a heterogeneous group of neoplasms with diverse biological behavior. Among malignant bone sarcomas, Ewing sarcoma (ES) is the second most common after osteosarcoma, predominantly affecting children, adolescents, and young adults [1]. Histologically, ES consists of small, round, blue tumor cells, while at the molecular level it is defined by characteristic chromosomal translocations, most frequently t(11;22)(q24;q12), which generate the EWSR1::FLI1 fusion gene responsible for aberrant transcriptional activation and oncogenic transformation [2]. ES primarily involves the axial skeleton and long bones, particularly the pelvis, femur, and ribs. Craniofacial involvement is rare, accounting for only 1-4% of cases, and maxillary involvement represents an exceptional subset. Its rarity, combined with its ability to clinically mimic benign odontogenic or inflammatory conditions, often contributes to delayed diagnosis [3].

The overall incidence of ES in the general population is estimated at approximately 0.1 cases per 100,000 individuals per year, with a peak incidence between 10 and 20 years of age and a slight male predominance [3]. This figure represents ES overall and does not specifically pertain to craniofacial or maxillary tumors, which are described in the literature as rare subsets of cases rather than as distinct population-based incidences. Several factors (including germline alterations in TP53 and RB1, environmental exposures, and accelerated bone growth during adolescence) have been associated with its development [4].

Accurate diagnosis of ES requires the integration of clinical, radiologic, histopathologic, and immunohistochemical findings. Diffuse membranous expression of cluster of differentiation 99 (CD99) and nuclear positivity for Friend leukemia integration 1 (FLI-1) are characteristic immunohistochemical features and, when interpreted within the appropriate clinicoradiologic context, strongly support the diagnosis. Molecular confirmation through identification of EWSR1 gene rearrangements represents the diagnostic gold standard; however, its availability may be limited in certain settings. In such cases, a comprehensive morphologic and immunophenotypic profile can be sufficient to establish the diagnosis. Negative staining for special AT-rich sequence-binding protein 2 (SATB2) and the absence of osteoid matrix further assist in distinguishing ES from other small round cell malignancies, such as mesenchymal chondrosarcoma and small-cell osteosarcoma [5].

The management of ES requires a multidisciplinary approach incorporating systemic chemotherapy and local control strategies, including wide-margin surgical resection and/or radiotherapy. Depending on tumor location, extent, and institutional protocols, chemotherapy may be administered in either the neoadjuvant or adjuvant setting. These strategies have increased five-year overall survival to nearly 70% in patients with localized ES, whereas outcomes remain poor in the presence of metastatic disease [6].

Maxillary ES presents distinct diagnostic and therapeutic challenges due to the complex anatomy of the midface, the need to balance oncologic control with functional and aesthetic preservation, and the potential for diagnostic uncertainty in settings with limited resources. This report provides clinical value by demonstrating the diagnostic process for a primary maxillary ES in a young adult woman managed without molecular confirmation and highlights a stepwise surgical approach guided by intraoperative margin assessment, incorporated within a multidisciplinary treatment plan that included systemic chemotherapy and adjuvant radiotherapy according to institutional protocols. It contributes to the limited literature on maxillary ES by emphasizing real-world diagnostic decision-making and functional rehabilitation within a public healthcare setting. This case is reported following the Surgical CAse REport (SCARE) 2025 guidelines [7].

Written informed consent was obtained from the patient for publication of this case report and accompanying clinical images.

## Case presentation

A 23-year-old female from Chalchihuites, Zacatecas, Mexico, with no significant past medical history, presented with a progressive, painless swelling of the anterior maxillary region. Her family history was notable for breast cancer in a maternal aunt. She denied chronic medical conditions, previous surgeries, drug allergies, or toxic habits. The patient initially noticed a gradual increase in maxillary volume without pain or systemic symptoms. After several months of progression, she sought dental care and was subsequently referred to the maxillofacial surgery service for further evaluation. The clinical course, including the timing of diagnostic and therapeutic interventions, is summarized in Table 1.

**Table 1:** Clinical timeline of the case (relative time from symptom onset)

Time from symptom onset	Clinical event/diagnostic step	Key findings
Month 0	Onset of symptoms	Progressive, painless enlargement of the anterior maxillary region
Months 3–4	Initial dental evaluation and referral to maxillofacial surgery	Progressive maxillary lesion prompting specialized assessment
Month 4	Incisional biopsy	Sarcomatous neoplasm initially interpreted as compatible with fibrosarcoma
Months 4–5	Histopathological reassessment	Undifferentiated malignant neoplasm; immunohistochemical analysis requested
Month 5	Imaging studies (facial bones, neck, and chest CT)	Aggressive maxillary bone lesion; no evidence of regional or distant metastatic disease
Month 6	Immunohistochemical confirmation	Diagnosis of Ewing sarcoma established (diffuse CD99 positivity with FLI-1 expression)
Month 6	Definitive surgical management	Subtotal maxillectomy performed with oncologic intent
Month 7	Postoperative histopathological examination	Ewing sarcoma with tumor-free surgical margins
Months 7–12	Adjuvant therapy	Systemic chemotherapy followed by complementary radiotherapy
Month 12	First post-treatment imaging follow-up	No evidence of local recurrence or systemic disease
Month 36	Long-term clinical follow-up	Patient asymptomatic, without clinical signs of disease recurrence
Month 42	Late imaging follow-up	Sustained absence of active disease on cross-sectional imaging

**Figure 1:**
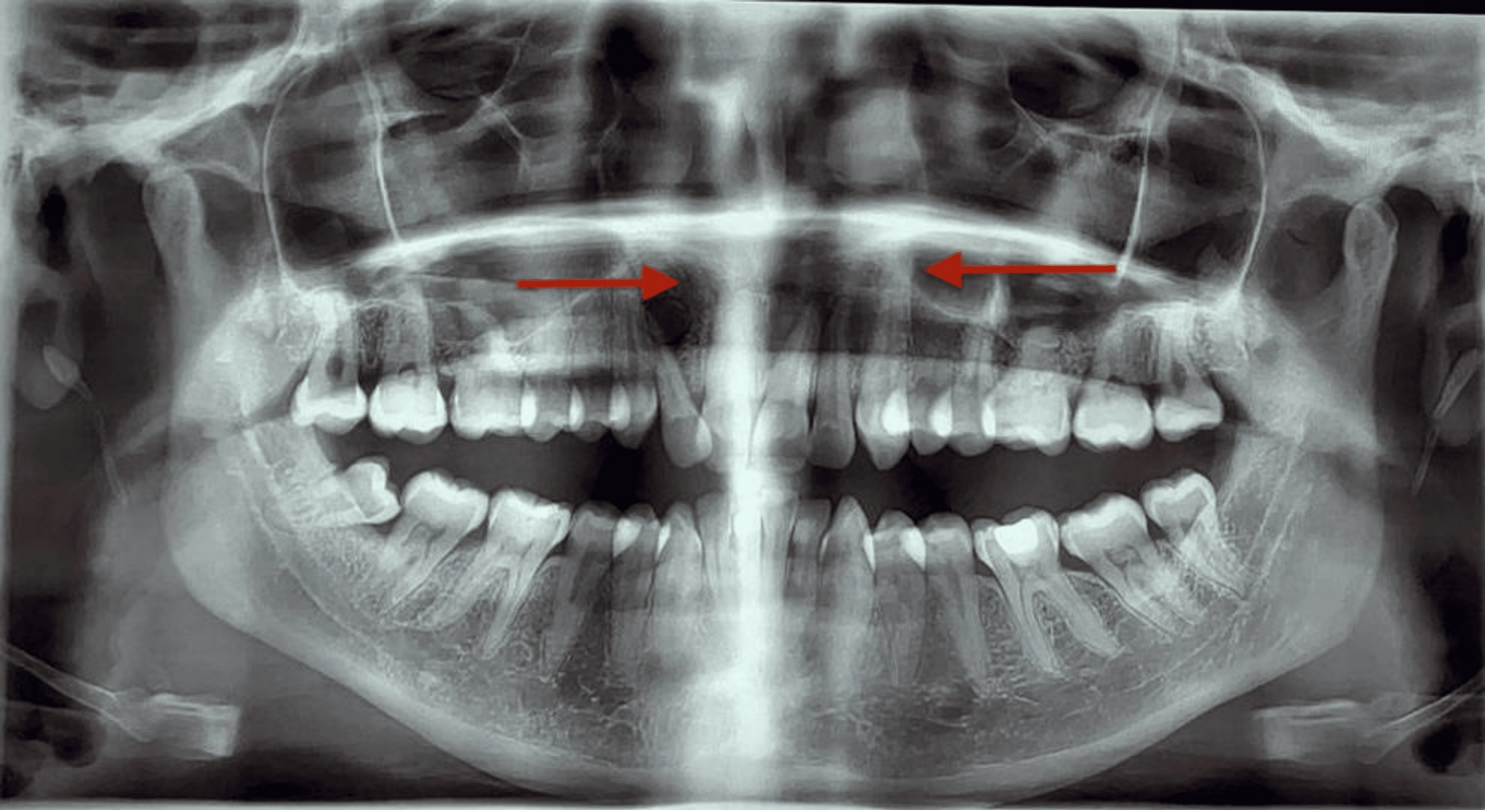
Initial panoramic radiograph The arrows indicate an extensive radiolucent lesion involving the anterior maxilla, extending across the midline, characterized by loss of the normal trabecular bone pattern and marked cortical thinning along the alveolar ridge, while the dentition remains intact. On panoramic radiography, no evidence of soft tissue involvement can be assessed. These findings are consistent with an expansile intraosseous lesion of the maxilla

Initial panoramic radiography demonstrated an extensive radiolucent lesion involving the anterior maxilla, extending across the midline, characterized by loss of the normal trabecular bone pattern and marked cortical thinning along the alveolar ridge. At the same time, the dentition remained preserved (Figure 1). No radiographic evidence of soft tissue involvement could be assessed on the panoramic view.

**Figure 2:**
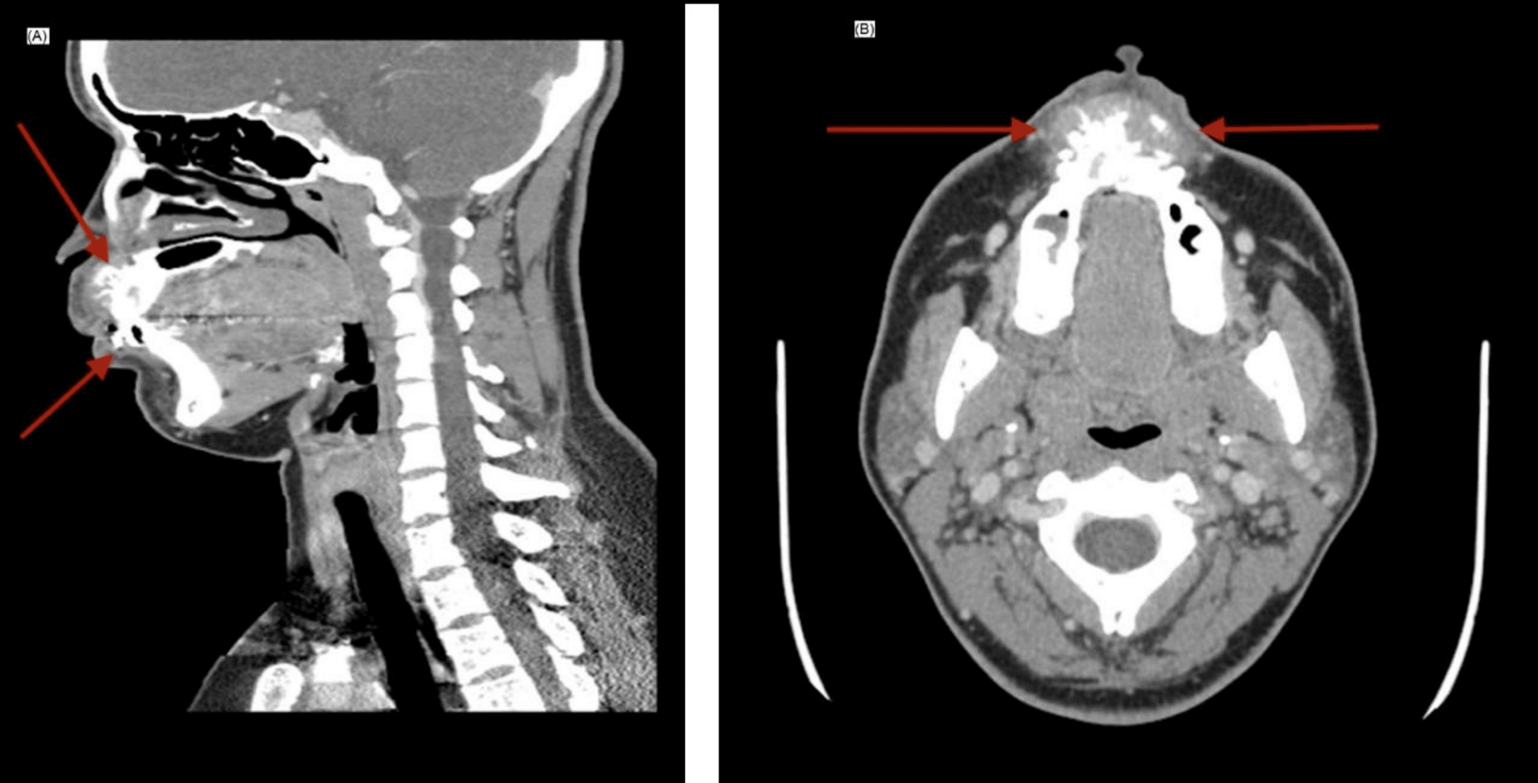
Contrast-enhanced CT of the facial bones (A) Sagittal reconstruction showing an expansile osteolytic lesion of the anterior maxilla with cortical erosion extending toward the nasal cavity. (B) Axial view demonstrating cortical destruction with adjacent soft tissue thickening of reactive appearance, without formation of a discrete extraosseous soft tissue mass or radiologic evidence of cervical lymph node involvement CT: computed tomography

CT of the facial bones revealed an expansile osteolytic lesion predominantly affecting the anterior maxilla, associated with cortical destruction and a spiculated periosteal reaction with a radiating “sunburst-like” appearance (Figure 2). Other classic periosteal patterns described in ES, such as laminated (onion-skin) reaction or Codman triangle, were not identified. Adjacent soft tissues demonstrated mild reactive thickening without the formation of a discrete extraosseous soft tissue mass, and no radiologically suspicious cervical lymphadenopathy was observed.

As part of the initial systemic staging workup before definitive local therapy, chest CT imaging was performed, and demonstrated no evidence of distant metastatic disease. Based on tumor size (<5 cm), absence of radiologic regional lymphadenopathy, and M0 status on available imaging, the case was classified as clinical stage IA according to the American Joint Committee on Cancer (AJCC) TNM staging system for bone tumors, as adopted by the National Comprehensive Cancer Network (NCCN) bone cancer guidelines; advanced staging studies (PET-CT/bone scan and bone marrow evaluation) were not available at our institution and are acknowledged as a limitation.

**Figure 3:**
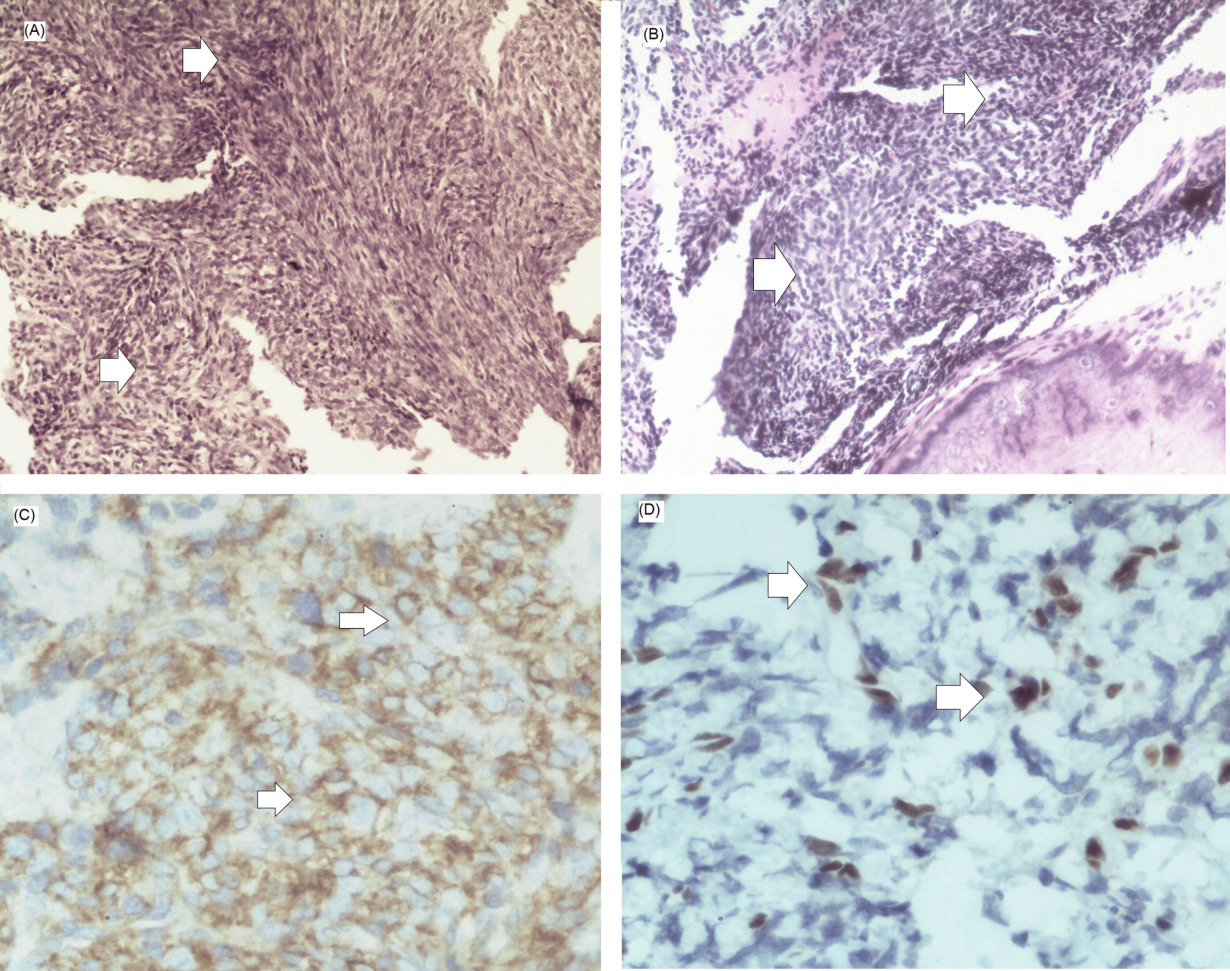
Histopathologic and immunohistochemical findings (A) H&E stain(hematoxylin and eosin, ×200). The arrows indicate sheets of uniform, small, round tumor cells, approximately 1–2 times the size of lymphocytes, with round nuclei, finely stippled chromatin, inconspicuous nucleoli, and scant, clear to eosinophilic cytoplasm. The cells exhibit indistinct cytoplasmic borders and a solid sheet-like growth pattern, characteristic of classical Ewing sarcoma. (B) H&E stain (×200). The arrows highlight lobules and nests of small, round, blue cells separated by dense fibrous septa, creating a pseudo-lobulated architecture. This panel also demonstrates areas of neuroectodermal differentiation, including Homer-Wright-type pseudorosettes, consistent with the spectrum of Ewing sarcoma morphology. (C) CD99 immunohistochemistry (×400). The arrows mark diffuse, strong membranous staining of the tumor cells, a hallmark immunophenotypic feature supporting the diagnosis of Ewing sarcoma. (D) FLI-1 immunohistochemistry (×400). The arrows show nuclear positivity in a subset of tumor cells, further supporting classification within the Ewing sarcoma family of tumors

An incisional biopsy was subsequently performed. The initial histopathological interpretation suggested a mesenchymal sarcoma compatible with fibrosarcoma of the anterior maxilla. A second histopathological review described an undifferentiated malignant neoplasm, prompting extended immunohistochemical analysis. Tumor cells demonstrated strong diffuse membranous positivity for CD99 (approximately 95%) and focal nuclear expression of FLI-1 in about 30% of cells. Immunostaining for S-100, vimentin, chromogranin, synaptophysin, CD45, cytokeratin AE1/AE3, desmin, myogenin, WT-1, and calretinin was negative, favoring a diagnosis within the ES family of tumors in the appropriate clinicoradiologic context (Figure 3). Molecular confirmation of EWSR1 gene rearrangements and NKX2.2 immunostaining were not performed due to limited diagnostic resources at our institution; therefore, the diagnosis was established based on an integrated assessment of clinical presentation, radiologic findings, histopathologic morphology, and immunohistochemical profile.

Definitive surgical management consisted of a subtotal maxillectomy with curative intent. Owing to the anatomical and aesthetic complexity of the midfacial region, where conventional 2-cm oncologic margins are often not feasible, the extent of resection was guided by intraoperative frozen-section analysis. Surgical margins were progressively extended until histological clearance was confirmed by the pathologist.

**Figure 4:**
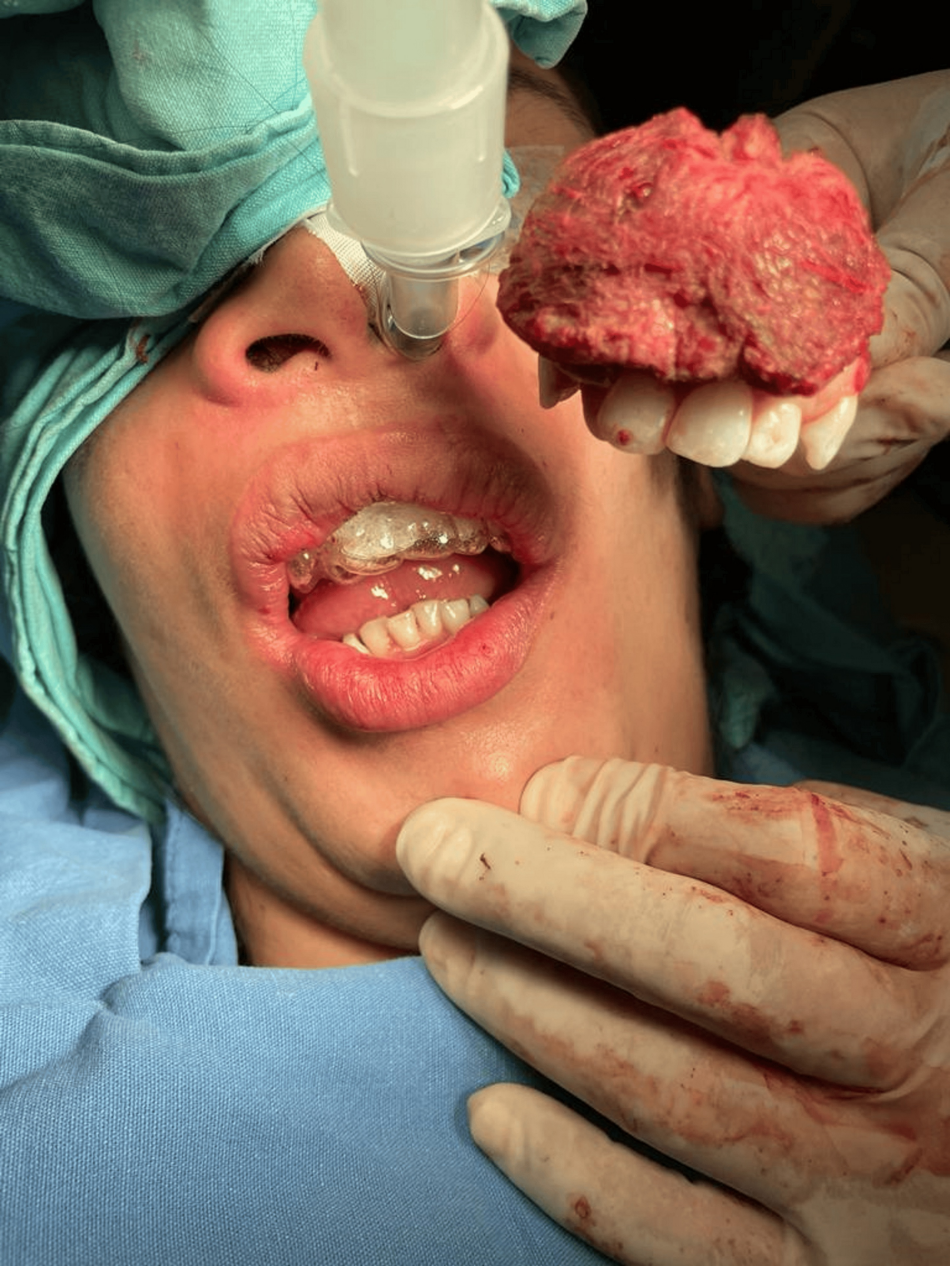
Intraoperative photograph during subtotal maxillectomy demonstrating the resected specimen corresponding to the anterior alveolar segment with a solid tumor mass

The resected specimen was oriented intraoperatively and submitted for pathologic evaluation with clear designation of the anterior (alveolar) and posterior aspects, medial (palatal) and lateral (buccal) surfaces, as well as superior (nasal floor) and inferior (alveolar ridge) margins, allowing accurate three-dimensional correlation between tumor extent and margin status. Gross examination revealed a solid, irregular mass involving the anterior alveolar segment and adjacent teeth (Figure 4). Final histopathological evaluation confirmed ES infiltrating the maxillary bone with extension into the gingival and premaxillary soft tissues. The tumor measured 4.5 × 2.8 × 2.5 cm, and all surgical margins were free of neoplastic involvement, with the closest deep margin measuring 0.3 cm.

The postoperative course was uneventful, with satisfactory wound healing and progressive functional recovery. No early postoperative complications were observed. Follow-up imaging demonstrated expected postoperative changes at the surgical site, without radiologic evidence of residual or recurrent disease. As part of a multimodal treatment strategy, the patient received adjuvant systemic chemotherapy followed by localized radiotherapy, administered according to institutional protocols for Ewing sarcoma.

During subsequent clinical and radiological follow-up, the patient remained asymptomatic, with preserved oral function and satisfactory aesthetic results on clinical evaluation. Given the extent of the anterior maxillary defect, functional rehabilitation was achieved through prosthetic obturation rather than primary bony reconstruction. The obturator restored separation between the oral and nasal cavities, allowing adequate speech and mastication and preventing oro-nasal communication. At follow-up, the patient demonstrated a Karnofsky Performance Status of 90% and an Eastern Cooperative Oncology Group (ECOG) performance status of 0. Serial imaging studies have shown no evidence of local recurrence or distant disease.

## Discussion

ES of the head and neck represents an exceptionally uncommon entity, accounting for approximately 1-4% of all ES cases, with maxillary involvement observed in less than 1%. Its clinical presentation is often nonspecific, frequently mimicking benign odontogenic, infectious, or inflammatory processes. In the present case, the patient developed a painless, progressively enlarging premaxillary mass, consistent with the observations of Shah et al. [8] and Mishra et al. [9], who reported maxillary lesions initially misdiagnosed as fibrosarcoma or odontogenic tumors, underscoring the diagnostic challenge posed by this anatomic region. The stepwise histopathologic evaluation in our patient reflects the diagnostic complexity of maxillary ES, which initially mimicked fibrosarcoma and undifferentiated sarcoma on routine hematoxylin-eosin (H&E) staining. Only after extended immunohistochemical profiling [showing diffuse CD99 and focal FLI-1 positivity with negativity for epithelial, neural, and myogenic markers] was the diagnosis established. This sequential process underscores the importance of correlating histology with immunohistochemistry in head and neck sarcomas, as reported by Sharma et al. [10] and Raghani et al. [11].

Histopathologically, ES is characterized by a proliferation of small round blue cells with diffuse membranous CD99 expression and nuclear FLI-1 positivity, which remain key diagnostic features when interpreted within the appropriate clinicoradiologic context. Molecular confirmation through detection of the EWSR1::FLI1 fusion transcript - present in approximately 85-90% of cases - is considered the diagnostic gold standard and is strongly recommended when available [8,12]. However, access to molecular testing may be limited in resource-constrained settings. In the present case, the diagnosis was established based on an integrated evaluation of clinical presentation, imaging findings, histopathologic morphology, and immunohistochemical profile, an approach consistent with that described by Raghani et al. [11] and Sharma et al. [10].

Although newer markers such as NK2 homeobox 2 (NKX2.2) have been shown to improve diagnostic specificity, particularly in distinguishing ES from other small round cell tumors, including lymphoma and rhabdomyosarcoma [12], these studies were not available at our institution. We acknowledge that the absence of molecular confirmation introduces a theoretical risk of diagnostic overlap or misclassification; however, this risk was mitigated through extensive immunohistochemical exclusion of competing entities and careful clinicoradiologic correlation. This limitation underscores the importance of comprehensive clinicopathologic correlation in settings where advanced molecular diagnostics cannot be routinely performed.

Regarding treatment, there is a broad consensus that optimal management of ES is multimodal, combining systemic chemotherapy with local control strategies that may include surgery and/or radiotherapy [9-11]. In the present case, the patient underwent subtotal maxillectomy with negative margins, followed by adjuvant systemic chemotherapy (epirubicin, cisplatin, and ifosfamide) and localized radiotherapy, achieving sustained local disease control at 18 months of follow-up. Although contemporary international guidelines commonly recommend vincristine, doxorubicin, and cyclophosphamide alternating with ifosfamide and etoposide (VDC/IE) regimens, treatment strategies may vary according to institutional protocols and drug availability. In our public hospital setting, the selected regimen reflected local practice and resource constraints and was implemented within a multidisciplinary framework. In contrast, Raghani et al. [11] reported a more conservative strategy involving curettage and neoadjuvant chemotherapy in pediatric cases, prioritizing facial growth and aesthetics. Importantly, neoadjuvant chemotherapy is also commonly employed in adult patients with ES to reduce tumor volume, assess chemosensitivity, and facilitate more conservative surgical approaches, with postoperative evaluation of residual disease. Such therapeutic variability highlights the importance of individualized management according to age, tumor extent, institutional context, and functional considerations.

Recent literature suggests that craniofacial ES is associated with a more favorable prognosis than axial or pelvic disease, with reported five-year survival rates ranging from 70 to 82% [11,12]. Key prognostic factors consistently identified include early diagnosis, smaller tumor size, and achievement of negative surgical margins, whereas radiologic response alone does not reliably predict outcome. In our case, effective local control was achieved through complete resection of a tumor measuring less than 5 cm, which likely contributed to the favorable clinical course observed at 18 months. Based on available staging studies, including a negative chest CT, the case fulfilled criteria for clinical stage IA disease. However, as advanced systemic staging studies were not performed, the presence of occult metastatic disease cannot be definitively excluded. Accordingly, the outcome should be interpreted as sustained local disease control rather than confirmed long-term systemic remission. Consistent with this, Mishra et al. [9] and Shah et al. [8] reported that complete resection remains the strongest predictor of local control, whereas partial radiographic response does not necessarily indicate poor prognosis if histologic necrosis is substantial. Similarly, Khan et al. [13] emphasized that negative margins and small tumor volume are among the most reliable indicators of a favorable outcome, and that incomplete radiologic response to chemotherapy does not preclude favorable results when oncologic resection is adequate.

Despite the favorable outcome observed in this case, it is important to acknowledge the limitations inherent to current intraoperative margin assessment strategies in head and neck oncology. Margin status has well-established prognostic implications, yet surgical decision-making is still largely based on gross visualization and manual palpation, supplemented by intraoperative frozen-section analysis. While this approach remains widely used and was appropriate in the present case, it has recognized limitations, including sampling error, difficulty in margin re-localization, and interobserver variability. Moreover, robust evidence directly linking frozen-section-guided margin assessment to improved survival outcomes remains limited.

Emerging technologies (such as three-dimensional specimen scanning, augmented reality guidance, optical and spectroscopic imaging, molecular margin analysis, and artificial intelligence-based tools) have shown promise in addressing these challenges. Although such technologies were not available in our institution at the time of treatment, their future integration into routine clinical practice may enhance margin accuracy, reduce the need for extensive resections, and further improve functional and aesthetic outcomes, particularly in anatomically constrained regions such as the maxilla [14]. Another relevant consideration is the difficulty in assessing chemotherapy response through imaging, as radiologic shrinkage does not always correlate with histologic necrosis [12]. This underscores the importance of multidisciplinary follow-up, integrating radiological, pathological, and laboratory assessments. Regular imaging every six months during the first two years is strongly recommended, as most recurrences occur within this period [10].

Accumulated evidence over the past decade demonstrates that maxillary ES managed with an integrated multidisciplinary approach can achieve sustained remission and satisfactory quality of life. The favorable functional and aesthetic outcomes in the present case are consistent with reports by Al-Bitar and Sandouk [12] and Mishra et al. [9], where prosthetic rehabilitation and psychosocial support were key elements of long-term care. Table 1 provides a descriptive overview of the limited number of reported cases of maxillary ES; however, factors such as the marked heterogeneity in patient age, tumor characteristics, diagnostic workup, treatment strategies, and duration of follow-up (often inconsistently reported) limit meaningful comparison of survival outcomes, recurrence rates, or prognostic equivalence across reports.

**Table 2:** Descriptive summary of reported cases of Ewing sarcoma involving the maxillary region (2022–2025) F: female; M: male; CT: chemotherapy; RT: radiotherapy; VDC/IE: vincristine, doxorubicin, cyclophosphamide/ifosfamide, etoposide; ES: Ewing sarcoma; NGS: next-generation sequencing; CK: cytokeratin; CD: cluster of differentiation (e.g., CD99, CD45); NKX2.2: homeobox protein NKX2.2; FLI-1: Friend leukemia integration 1 transcription factor

Author/year	Age in years/sex	Anatomical location	Initial clinical findings	Diagnostic confirmation	Treatment performed	Outcome/Follow-up	Distinctive features
Mishra et al., 2022 [9]	22/F	Mandible	Painless swelling, initially diagnosed as an odontogenic lesion	CD99+, FLI-1+, small round cells	Segmental resection + adjuvant CT	Disease-free at 24 months	Multilocular lesion; initially misdiagnosed
Raghani et al., 2023 [11]	13/M	Maxilla	Swelling with nasal obstruction resembling sinusitis	CD99+, FLI-1+, no translocation confirmed	Neoadjuvant CT (VDC/IE) + curettage + adjuvant CT	Disease-free at 2 years	Conservative pediatric approach; facial preservation
Sharma et al., 2024 [10]	12/F	Maxillary sinus	Facial pain and swelling with orbital extension	CD99+, typical ES histology	CT + RT (no surgery)	Local control; no recurrence at 18 months	Radiosensitive tumor; nonsurgical management due to intracranial extension
Khan et al., 2025 [13]	19/M	Posterior maxilla	Expansile lesion with dental displacement	CD99+, NKX2.2+, FLI-1+, no molecular fusion	Radical surgery + adjuvant CT	Disease-free at 12 months	NKX2.2 markers; emphasis on negative margins
Al-Bitar and Sandouk, 2025 [12]	14/F	Upper maxilla	Pain and swelling for 7 months	CD99+, NKX2.2+, FLI-1+, no fusion demonstrated	Neoadjuvant CT (VDC/IE) + en bloc resection + adjuvant CT	Complete remission at 12 months	Includes dental rehabilitation and psychosocial support
Shah et al., 2025 [8]	21/M	Right maxilla	Expansile lesion with tooth mobility and vestibular obliteration	CD99+, vimentin+, CK–, CD45–	Surgery + CT + RT	No follow-up reported	Highlights use of NGS to confirm EWS-ETS fusion
Present case	23/F	Anterior maxilla	Painless, slow-growing swelling	CD99+ (95%), FLI-1+ (30%)	Subtotal maxillectomy + CT (epirubicin, cisplatin, ifosfamide) + RT	Disease-free at 18 months	Early diagnosis, negative margins, multidisciplinary management

## Conclusions

ES of the maxilla is an uncommon malignant neoplasm whose diagnosis requires a high index of suspicion and systematic integration of clinical, radiologic, and immunohistochemical findings. Early and accurate identification allows planning of a multimodal therapeutic strategy combining oncologic resection, systemic chemotherapy, and adjuvant radiotherapy. Even in anatomically complex subsites such as the midface, complete excision with negative margins remains a central determinant of effective local disease control. This report illustrates how a coordinated multidisciplinary approach can achieve sustained local remission in clinically localized disease while maintaining satisfactory postoperative function and aesthetics. In our patient, functional rehabilitation with prosthetic obturation allowed preservation of speech and mastication, with a favorable performance status documented on clinical follow-up. Prognostic features such as early presentation, limited tumor size, negative surgical margins, and classification as clinical stage IA based on available staging studies likely contributed to the favorable clinical course observed at 18 months. Ongoing clinical and radiologic surveillance remains essential, particularly during the first two years, when the risk of recurrence is highest.
